# Stepping Up Enhanced Rate Calculations with EATR-Flooding

**DOI:** 10.1021/acs.jctc.6c00883

**Published:** 2026-07-29

**Authors:** Nicodemo Mazzaferro, Willmor J. Peña Ccoa, Pilar Cossio, Glen M. Hocky

**Affiliations:** † Department of Chemistry, 5894 New York University, New York, New York 10003, United States; ‡ Center for Computational Mathematics, 525571 Flatiron Institute, New York, New York 10010, United States; § Center for Computational Biology, Flatiron Institute, New York, New York 10010, United States; ∥ Simons Center for Computational Physical Chemistry, New York University, New York, New York 10003, United States

## Abstract

Several recent methods have shown that it is possible to compute rate constants of very slow biomolecular processes using simulations where a time-dependent bias is added along one or several collective variables (CVs). We previously reported the exponential average time-dependent rate (EATR) method, which can improve upon these approaches by accounting for how efficiently the external biasing potential modifies the observed rate using a learned CV-quality factor γ. This results in more accurate rate estimates using the same data when biasing a suboptimal coordinate. As formulated, however, EATR depended on the biasing potential varying over time to properly determine the biasing efficiency, which limits the method’s applicability to quasi-static biasing schemes such as “flooding” or on-the-fly probability enhanced sampling (OPES). Here, we present the EATR-flooding approach, which generalizes our method by replacing the need for a time-dependent bias by instead varying (stepping up) the strength of the biasing potential across multiple sets of simulations. We implement this approach in an open source Python library, and demonstrate that this approach is accurate without substantial loss of efficiency compared to standard EATR for a coarse-grained protein system, and also show good performance on a fully atomistic cavity-ligand model. Two additional appealing features of EATR-flooding are an internal check for overbiasing and the fact that only a single γ parameter is predicted for a given choice of CVs, as compared to our earlier results where the estimated γ empirically varied with the biasing rate. Finally, we believe EATR-flooding applies not only to OPES simulations but more generally to CV biasing enhanced sampling approaches, making it broadly useful.

## Graphical abstract

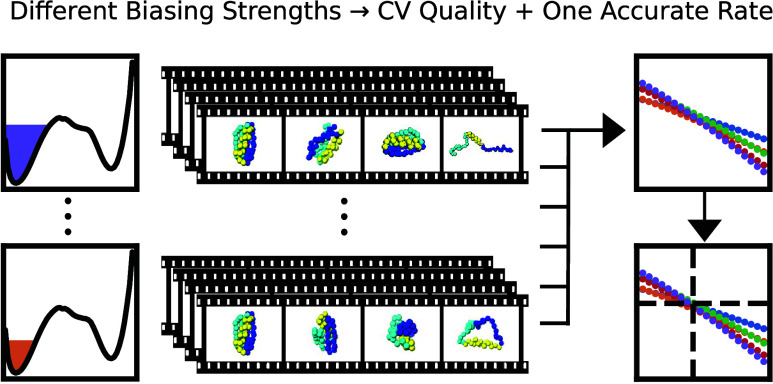

## 1 Introduction

The development of computational approaches to predict protein–ligand binding kinetics is an active area of research, due to the growing recognition of the importance of drug lifetime in controlling the efficacy and durability of pharmacological treatments.^1–6^ Classical molecular dynamics (MD) simulations provide a powerful approach to predict the behavior of biomolecules in solution as well as their interaction with ligands or other biomolecules.^7,8^ Despite the name, MD simulations are more often used to estimate short time scale properties of a system by integrating the equations of motion to produce statistical samples of the system at fixed temperature or fixed temperature and pressure (distorting the microscopic kinetics), rather than long-time scale dynamical properties.^9^ For many biophysical problems of interest, however, the dynamics of the most relevant configurational changes are dominated by transitions over free-energy barriers between metastable states, meaning the kinetics can still be computed by statistical approaches.

Therefore, atomistic MD simulations can still provide a powerful technique for predicting the time scale or average rate constant for transitions between these states, as long as sufficient transitions can be observed within an accessible computational time. Unfortunately, there is often a substantial difference in time scales between what can be accessed in MD simulations as compared to the time scales of the events of interest. Modern simulations often generate microseconds of trajectory data for relatively simple systems, whereas the processes of biomolecular interest occur on time scales ranging from milliseconds to seconds. The transitions of interest are so-called rare events, in the sense that the system spends long periods in a metastable state before crossing into a new state in a relatively fast manner. Because of this undesired tendency to remain in a single region of state space for long periods of time, a number of enhanced sampling methods have been developed to quickly drive systems to escape free-energy minima by adding an extra bias along certain collective coordinates or variables (CVs) describing the system.^9–15^ This extra bias effectively lowers the barrier to transitions, allowing one to reconstruct the underlying free-energy landscape through inverting the effect of the bias on the frequency of observed configurations. The two methods that we focus on in this work are Metadynamics (MetaD) and On-the-fly probability enhanced sampling (OPES), which both work by constructing a bias on the fly which is tailored to push the system away from already explored regions of CV space.^12–14,16^

In both approaches, the consequence of adding this external bias is to significantly and intentionally reduce the long dwell times before the transitions. However, it is not always clear that the rate constants of interest can be recovered under these biases in the same way as thermodynamic properties. Several methods have been developed to recover kinetics from CV biasing techniques, relying on using a thermodynamic model for the kinetic process of interest, such as Kramers theory.^17–24^ In essence, doing so requires assuming that the bias is applied only to destabilize the starting state and does not directly affect the transition over the barrier, in which case transition times are sped up approximately by the average of the exponential of the applied bias. For example, in Infrequent Metadynamics (iMetaD),^18^ this is achieved by only updating the biasing potential on a time scale longer than the barrier crossing time. In OPES-flooding (and also the earlier Variationally Optimized Free-Energy Flooding^20^ based on Variationally Enhanced Sampling^15^), this is achieved by capping the highest level of bias applied to the system to fall below a previously estimated barrier level (Δ*E*), and also by defining an excluded region where the bias is rigorously set to zero.^22^ All of these methods also have an implicit assumption that the bias is applied to a CV that is a “good” reaction coordinate for the process of interest, since bias applied perpendicular to the ideal reaction coordinate should not be included in estimating such a speedup factor. Despite these stringent requirements, iMetaD and OPES-flooding shine in the context of giving a computationally tractable way to estimate kinetics of very slow biomolecular processes (milliseconds to days) using only nanoseconds or microseconds of total simulation.^23^

In our work, we have concentrated on relaxing the requirement to choose a good CV, since in practice this is challenging to do *a priori* - or even *a posteriori*. We have pursued a strategy whereby we extract the rate constant from the data along with an additional parameter γ that takes into account the efficiency of the chosen CV for producing desired transitions.^25,26^ In our Exponential Average Time-dependent Rate (EATR) approach—described in detail in the next section—we estimate both the rate and γ by averaging our estimate of the speedup factor across many MetaD simulations, and then fitting transition times either via maximum likelihood or by fitting the cumulative distribution of observed transition times.

While EATR worked well for simulations biased with MetaD, it failed when applied to simulations biased with OPES.^26^ This failure was due to the rapid convergence of OPES to a final biasing function in the starting basin, which prevents our fitting approach from disambiguating the unbiased rate from the estimate of CV quality, as explained below. To solve this, we propose a new method called EATR-flooding which estimates γ using the biased rates from multiple sets of simulations, each with a different average amount of bias applied before transitions are observed; in OPES this is achieved by varying the value of Δ*E*. By “stepping up” the value of this parameter, which limits the average amount of bias that is added, we are able to probe the dependence of the observed rate on the amount of bias added, which can allow us to extract information about the quality of the biased CV(s) with minimal extra cost. We first speculated about this approach in the Supporting Information of our earlier study^26^ and showed how by varying the amount of bias, we could, with an uncontrolled approximation, extract an approximate rate and γ. In this article, we put this method on more rigorous grounds. We then test our new method on a coarse-grained model for protein folding as well as a fully atomistic model of a ligand binding problem, and show that it provides efficient and accurate estimates for the unbiased rate, using a single γ parameter to account for the quality of the biased CV. We also show that the method generalizes beyond OPES, working for the case of a fixed external bias as originally envisioned in the conformational flooding or hyperdynamics methods,^17,27^ and also for a time-dependent bias applied with iMetaD.

The remainder of this paper is organized as follows. In Section 2, we summarize the previously studied methods relevant to this article. In Section 3, we explain the necessity for and the theory behind the EATR-flooding method. In Section 4, we present the results of applying the EATR-flooding method to a coarse-grained protein and model cavity-ligand system. In Section 5, we present our conclusions from the results. Finally, in Section 6, we provide details of our computational methods.

## 2 Background

### 2.1 On-the-Fly Probability Enhanced Sampling

OPES is an enhanced sampling technique that adds an additional potential energy term *V*(ξ) called a *biasing potential*, or simply the *bias*, which depends on a CV ξ. The biasing potential at time *t* is given by

1 $$V_{t}\left(\xi \right)=\left(1-1/B\right)\frac{1}{\beta }\log\left(\frac{P_{t}\left(\xi \right)}{Z_{t}}+\epsilon \right)$$

where *B* is the “bias factor,” *P*_*t*_(ξ) is the estimate at *t* of the probability density, *Z*_*t*_ is a normalization constant for *P*_*t*_(ξ), and ϵ = *e* ^–*β*Δ*E*/(1–1/*B*)^ is a regularization term included to hinder the bias from increasing past a preset amount of energy Δ*E*, termed BARRIER in the PLUMED open source sampling library,^28,29^ as this is useful to keep the bias energy below an energy barrier for kinetics calculations.

In OPES, it is possible to enforce that bias is not applied when ξ is within a predefined area, set by defining an “excluded region.” This can be useful for kinetics calculations, since this makes it possible to prevent bias from being added to CV values believed to capture the transition state.^22^ We do not necessarily need to use the excluded region for our calculations in this work, and indeed we did not use it for our more challenging atomistic system below.

OPES is particularly efficient in generating an estimate of the probability density due to the fact that *V*(ξ) converges rapidly. For the OPES-flooding scenario where biasing is limited to the starting basin, after a short period at the beginning of the sampling, the bias remains effectively constant in time.

### 2.2 Infrequent Metadynamics and OPES-Flooding

iMetaD is a rate estimation technique inspired by hyperdynamics and conformational flooding.^18,27,30^ For this method, a biasing potential is constructed as a function of a CV ξ using MetaD, where a Gaussian of a specified height and width is added to the potential at a specified interval during an MD simulation. Several of these MetaD simulations are run until the transition occurs, and the transition time is rescaled according to

2 $$\tau_{i}=\int_{0}^{t_{i}}e^{\beta V\left(\xi \left(t\prime \right),t\prime \right)}dt^{\prime }$$

where τ_*i*_ and *t*_*i*_ are the rescaled and observed transition times from simulation *i*, respectively.^18^ This is often expressed as τ_*i*_ = α_*i*_ *t*_*i*_, where

3 $$\alpha_{i}=\frac{1}{t_{i}}\int_{0}^{t_{i}}e^{\beta V\left(\xi \left(t\prime \right),t\prime \right)}dt^{\prime }$$

α is called the acceleration factor.

With the rescaled times, it is possible to estimate the rate, either by computing the inverse of the mean first passage time or by fitting the Poisson process cumulative distribution function (CDF) *h*(τ) = 1 – *e* ^–*k*_0_τ^, using the rescaled times τ_*i*_.^19^

OPES-flooding (OPESf) follows the same principle as iMetaD but applies it to simulations biased using OPES instead of MetaD.^22^ This approach could offer advantages due to the ability to have finer control over the amount and location of the added bias, as described in the previous section.

### 2.3 Exponential Average Time-Dependent Rate

Both iMetaD and OPESf assume an ideal reaction coordinate such that all bias goes into accelerating the transitions. For iMetaD, we previously reported on the KTR approach^25^ and EATR method,^26^ which allow us to compute more accurate rate constants from iMetaD trajectories by accounting for the fact that we are not biasing an ideal reaction coordinate. Here, we focus on EATR, which we demonstrated is a better ansatz, although KTR may still work equally well in real applications.

In EATR, the instantaneous rate constant observed in the biased simulations is assumed to be related to the unbiased rate constant by the relationship

4 $$k\left(t\right)=k_{0}\overset{®}{e^{\beta \gamma V\left(\xi \left(t\right),t\right)}\;}$$

where *k*(*t*) is the biased rate at time *t*, *k*_0_ is the unbiased rate, β = 1/*k*_B_ *T* is the inverse thermodynamic temperature, *V*(ξ­(*t*),*t*) is the biasing potential evaluated at time *t* for the value of the collective variable ξ­(*t*), and γ ∈ [0, 1] is the biasing efficiency measure from KTR. The overline represents an arithmetic mean over all simulations which did not transition before time *t*.

Because the rate constant is time-dependent, we model the transition as a nonhomogeneous quasi-static Poisson process with the survival probability

5 $$S\left(t\right)=e^{-\int_{0}^{t}k\left(t\prime \right)\mathrm{dt}\prime }=e^{-k_{0}\int_{0}^{t}\overset{®}{e^{\beta \gamma V\left(\xi \left(t\prime \right),t\prime \right)}\;}\mathrm{dt}\prime }$$

If we perform *N* biased simulations, where the first passage time of simulation *i* is *t*_*i*_, we can construct an empirical CDF $ĥ\left(t\right)=\frac{1}{N}\sum_{i=1}^{N}\Theta \left(t-t_{i}\right)$ where Θ­(*t*) is the Heaviside step function. The theoretical CDF, *h*(*t*) = 1 – *S*(*t*), can be fit to *ĥ*(*t*) with the parameters *k*_0_ and γ. The best-fit values of these parameters give us an estimate for the unbiased rate and the biasing efficiency, respectively.

If the biasing potential is constant in time, the simulation average in eq 4 is approximately the ensemble average:

6 $$\overset{®}{e^{\beta \gamma V\left(\xi \left(t\right)\right)}\;}\approx \left\langle e^{\beta \gamma V\left(\xi \right)}\right\rangle \equiv \alpha_{\gamma }$$

which is constant. In this case, the transition becomes Poissonian:

7 $$S\left(t\right)=e^{-k_{0}\int_{0}^{t}\left\langle e^{\beta \gamma V\left(\xi \right)}\right\rangle \mathrm{dt}\prime }=e^{-k_{0}\alpha_{\gamma }t}$$

and there is not a unique combination of *k*_0_ and γ consistent with the data, since any decrease to α_γ_ can be compensated for by an increase in *k*_0_. As such, EATR as previously formulated struggles to properly estimate *k*_0_ and γ when employing a static (or quasi-static) biasing potential, such as that used in OPES.

## 3 EATR-Flooding

In order to obtain estimates for *k*_0_ and γ using OPES, we need a formalism which does not assume that the biasing potential is time-dependent. We can do this by first applying eq 6 to eq 4, taking the logarithm on both sides, and solving for the logarithm of the estimated rate constant, yielding

8 $$\ln k_{\mathrm{est}}\left(\gamma \right)\equiv \ln k_{\mathrm{obs}}-\ln\left\langle e^{\beta \gamma V\left(\xi \right)}\right\rangle$$

where *k*_est_ is the estimate for *k*_0_, and *k*_obs_ is the constant value of the biased rate *k*(*t*). Note that this rate estimate depends on γ.

Given that there is only one true value of ln *k*_0_, we hypothesize that there exists a corresponding unique value for γ* which produces only one value of ln *k*_0_^*^. Therefore, we propose to determine γ* and *k*_0_^*^ by comparing the results of multiple *sets* of simulations, each with a different amount of bias, as shown in Figure 1a, and computing the effect of this different amount of bias on the observed rate constant. The unbiased rate for each set is estimated as a function of γ using eq 8, then the unbiased rate and γ estimates for the full collection of simulation sets are obtained by finding the value of γ where all sets of simulations have approximately the same unbiased rate estimate, or equivalently,

9 $$\gamma^{*}={\mathrm{argmin}}_{\gamma }\mathrm{Var}\left(\ln k_{\mathrm{est}}\left(\gamma \right)\right)$$

when the variance of the estimates across the sets is minimized. The best estimated value of the true rate is then $k_{0}^{*}=\overset{®}{k_{\mathrm{est}}\;\left(\gamma^{*}\;\right)\;}$ , where the average is done over the simulation sets. This is illustrated in Figure 1b–d.

**Figure 1:**
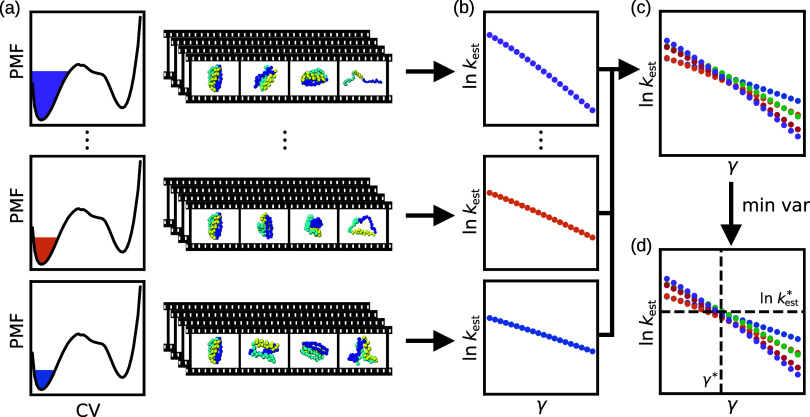
Schematic diagram for the EATR-flooding (EATRf) method. (a) Multiple sets of simulations are run until a transition occurs, where each set is run with a different average amount of applied bias, controlled by Δ*E* in OPES. Sets are color-coded for clarity. (b) Estimated log-rate for the transition is computed as a function of γ, ln *k*_est_ = ln *k*_obs_ – ln ⟨*e* ^*βγV*(ξ)^⟩. (c) Rate estimates are combined for all sets, and (d) the log-rate and γ which minimize the variance over the sets are taken to be the EATRf estimates.

We note that this approach does not require that the bias potential is static or quasi-static, but only that it is possible to converge an acceleration factor α_γ_ for each set of biasing parameters. Our EATRf method is therefore based on the same ansatz as the original EATR approach but is more general.

## 4 Results and Discussion

### 4.1 Coarse-Grained Model for Protein Folding

To evaluate our hypothesis in the preceding section, we first test our approach on a system sufficiently complicated that we can bias a wide range of CVs to obtain kinetic information, but sufficiently simple that we can obtain an unbiased rate constant estimate. We therefore adopted the same system used in our prior development of EATR, a Go̅-like model of the B1 domain of protein G, which had previously been established as a useful system for studying the quality of CVs for describing protein folding.^26,31,32^ As described in the Methods, we ran MD simulations where an OPES bias was applied to one of the following CVs: the fraction of native contacts *Q*, the end-to-end distance *R*_ee_, and the radius of gyration *R*_g_. We also performed simulations in which we biased *R*_ee_ and *R*_g_ simultaneously.

For each choice of CV, we ran five sets of simulations where we varied the maximum level of OPES bias, Δ*E*. This resulted in a different biasing potential in each set. As an example of how this looks when projected along two of the CVs, in Figure 2 we plot the average final bias obtained from OPES on top of the underlying PMF computed in our previous work.^26^ As can be seen, the amount of bias/forcing increases in concert with the Δ*E* parameter, and depends on the choice of CV. In Figure S1, we show that the effective average bias quickly plateaus to a static value, showing why we are not able to apply our original EATR method.

**Figure 2:**
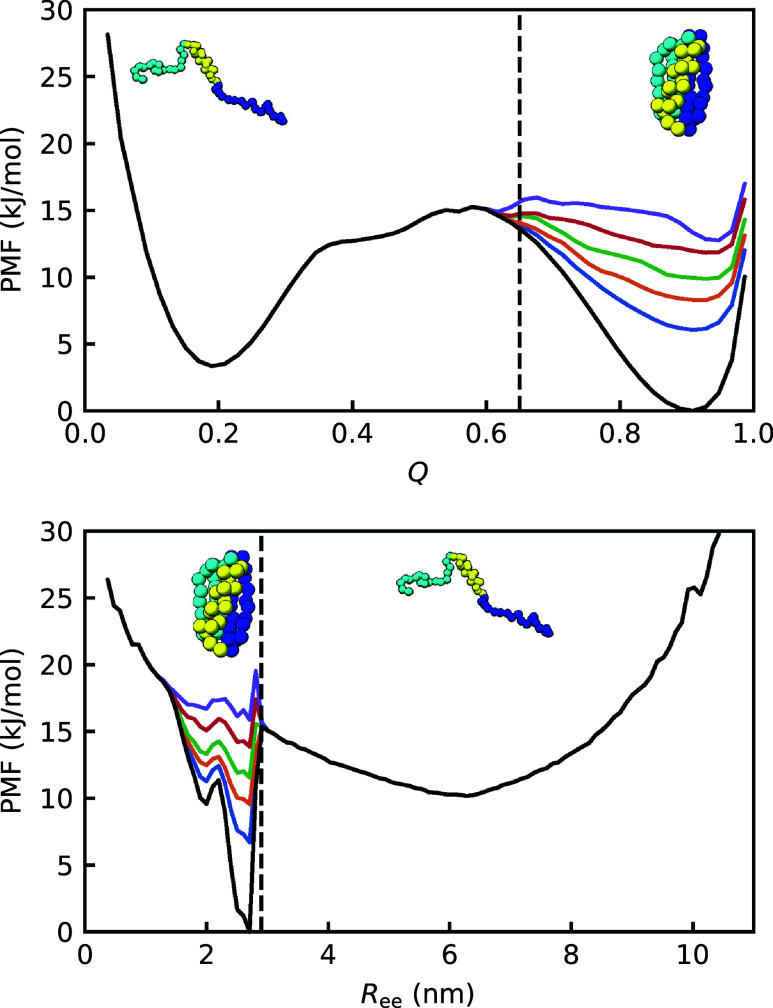
Potential of mean force (PMF) along the fraction of native contacts CV *Q* (top) and the end-to-end distance CV *R*_ee_ (bottom). The colored lines indicate the PMF with the average final biasing potential from OPES with various values of the Δ*E* parameter. From bottom to top, Δ*E* = 5, 7, 9, 11, and 13 kJ/mol. The vertical dashed lines represent the boundary of the excluded region.

We therefore proceed to apply eq 8 to these data while varying γ over the range 0–1, which gives us the results in Figure 3a. In this figure, the horizontal dashed line shows the log of the unbiased rate computed previously.^26^ With these data, we can directly see that the curves for each value of Δ*E* cross near the true rate. The crossover behavior can be explained as follows: for γ = 0, simulations with high Δ*E* are on top, which is simply a reflection of having faster observed transition times when the system is biased more strongly; in contrast, γ = 1 is equivalent to iMetaD which overpredicts transition times for nonideal CVs^26,33^ and consequently larger overpredictions (lower rate constants) arise for higher Δ*E*.

**Figure 3:**
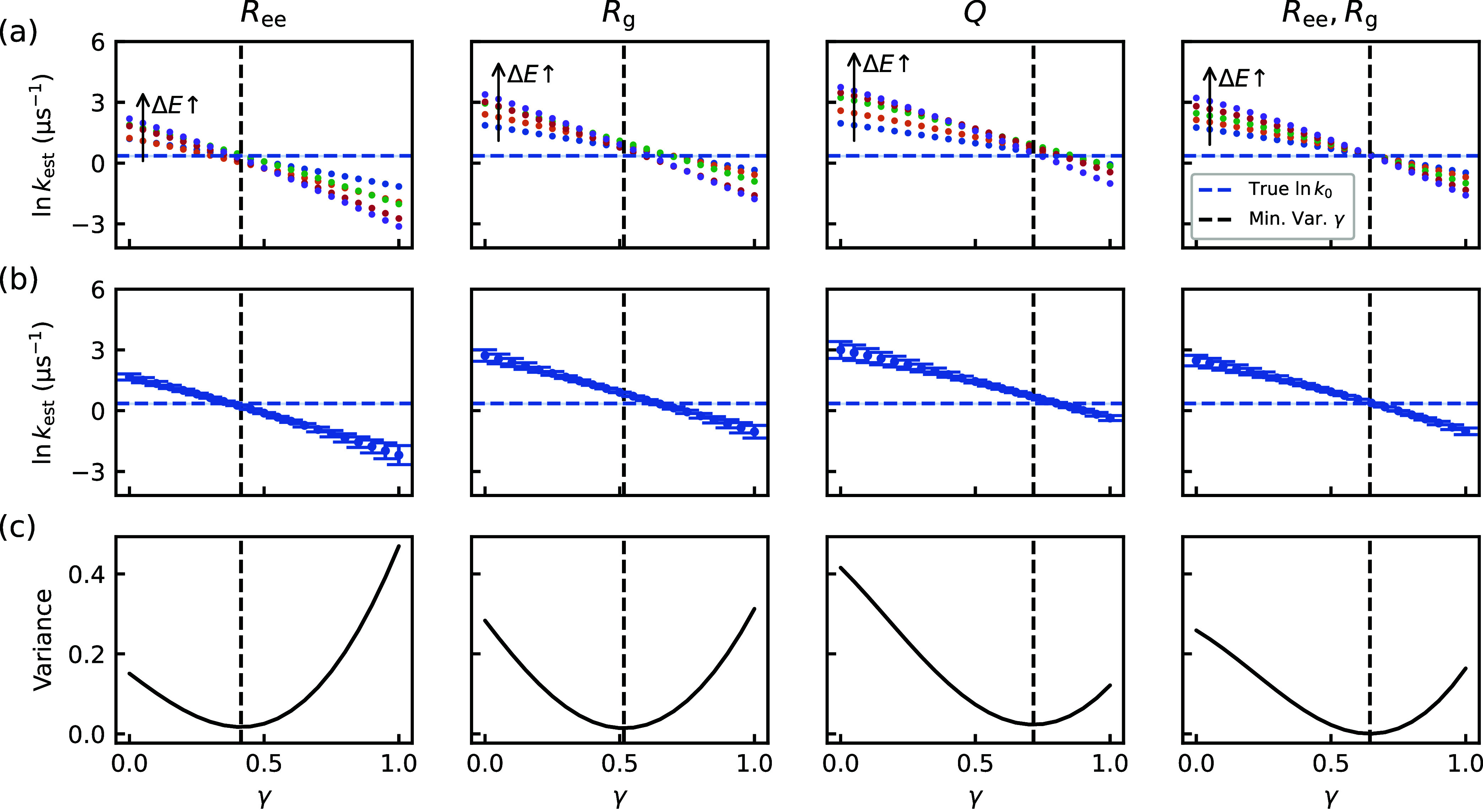
Results of EATR-flooding on the protein G model. (a) Values of ln *k*_est_ predicted by eq 8 for each set of OPES simulations with each Δ*E* parameter for various values of γ. (b) Value of ln *k*_est_ averaged over the sets of simulations for each potential value of γ. The variance of ln *k*_est_ across the sets is represented by error bars. (c) Variance in the predicted value of ln *k*_est_ across the sets of simulations. The horizontal dashed lines represent the reference value of ln *k*_0_, the uncertainty in which is too small to represent in this plot. The vertical dashed lines represent the minimum variance value of γ, which is the value that causes all sets to give the same value of ln *k*_est_.

In Figure 3b,c, we show the average and variance across data sets obtained from the values computed via eq 8. With a vertical dashed line, we indicate the γ, which has the lowest variance, as computed using an optimization algorithm as described in the methods. Visually, we can observe that the minimum variance occurs close to when the estimated log-rate value intersects that of the unbiased rate estimate.

Inspecting the minimum-variance γ values, we find that they are similar to those computed using our original EATR approach using data from iMetaD.^26^ As expected, *Q* has the highest value of γ while *R*_ee_ has the lowest. Simultaneously biasing the two lower quality CVs, which individually have γ values of 0.41 and 0.52, results in a combined γ value of 0.64, showing the synergistic value of multidimensional biasing. Of note, the γ value for *Q* is somewhat less for these OPES simulations than we observed with iMetaD, where γ could approach 1 for slow biasing along *Q*, which may reflect an interesting difference between how OPES and iMetaD build up the bias histogram, which is worthy of further investigation in the future.

In addition to this γ analysis, we can also see the quality of the CVs visually by plotting the log of the observed rate versus the log acceleration factor, ln⟨*e* ^*βV*^⟩, as shown in Figure S2. Since the OPESf acceleration factor is accurate for good CVs such as *Q*, we expect a linear relationship with near unit slope between these two terms. As discussed in ref 26, lower slopes indicate a bad or inefficient CV. This is discussed more in the next section.

Having demonstrated that EATR-flooding is an accurate approach, we then wanted to investigate whether the additional cost of running with multiple values of Δ*E* made the method prohibitively expensive compared to OPESf. To do so, we recomputed the predicted rates by EATRf using subsampling with smaller numbers of simulations per batch. As shown in Figure 4, the typical predicted rate constant is unchanged even when using only 10 simulations per batch, corresponding to a factor of 10 less simulations than used for the full analysis. However, the typical error in doing so is likely to be larger, and so there is some benefit to obtaining more data. From this analysis, we directly observe that the OPESf estimator is systematically incorrect for poor CVs, and additional sampling does not alleviate the issue. In contrast, the mean predicted value of *k*_0_ from EATRf is generally close to the unbiased value for all CVs, always within a factor of 2, and seems to have near perfect accuracy for 2D biasing of *R*_g_ and *R*_ee_ (see also Table 1).

**Table 1:** Rates and γ Values Obtained for the Protein G Model by Applying EATR-Flooding to Different CVs, Compared to the Rates Obtained from Conventional OPES-Flooding, Combining Data from All Sets with the Δ*E* Values 5, 7, 9, 11, and 13 kJ/mol^a^

OPES-flooding	
CV	ln *k*_0_^est^ (with *k*_0_ in μs^–1^)
*R*_ee_	–1.92 ± 0.07
*R*_g_	–0.82 ± 0.05
*R*_g_, *R*_ee_	–0.63 ± 0.05
*Q*	0.02 ± 0.05

EATR-flooding (OPES)		
CV	ln *k*_0_^est^ (with *k*_0_ in μs^–1^)	γ
*R*_ee_	0.23 ± 0.23	0.41 ± 0.06
*R*_g_	0.85 ± 0.16	0.52 ± 0.04
*R*_g_, *R*_ee_	0.42 ± 0.17	0.64 ± 0.05
*Q*	0.62 ± 0.19	0.74 ± 0.06

EATR-flooding (fixed bias)		
CV	ln *k*_0_^est^ (with *k*_0_ in μs^–1^)	γ
*R*_ee_	0.40 ± 0.18	0.31 ± 0.07
*Q*	0.53 ± 0.16	0.88 ± 0.07

EATR-flooding (iMetaD)		
CV	ln *k*_0_^est^ (with *k*_0_ in μs^–1^)	γ
*R*_ee_	0.43 ± 0.06	0.28 ± 0.01
*Q*	0.47 ± 0.17	0.67 ± 0.06

**unbiased ln *k*_0_ (with *k*_0_ in μs^–1^)**	0.36 ± 0.07

a The “unbiased” entry is obtained from running 100 equilibrium MD simulations, from ref 26.

**Figure 4:**
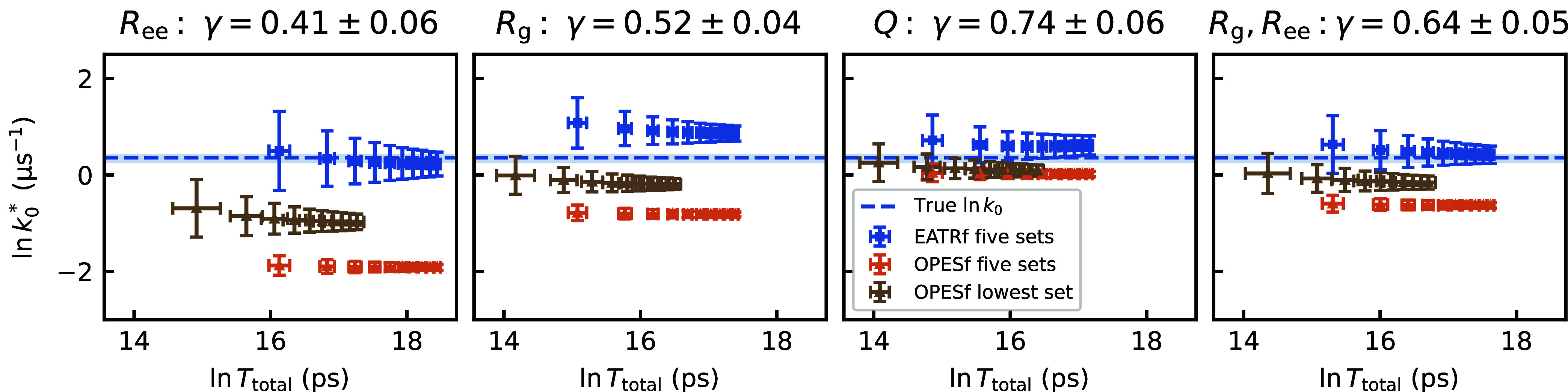
Predicted value of ln *k*_0_ from EATR-flooding (in blue) and OPES-flooding (in orange) as a function of the logarithm of the total simulation time with the lowest five values for Δ*E*. Various numbers of simulations per set, from 10 (left side of each plot) to 100 (right side of each plot). The blue dashed line represents the rate obtained from unbiased simulations. Error bars indicate the standard error in bootstrap analysis.

As just discussed, Figure 4 shows that our approach of combining data from multiple sets of simulations biased by different amounts is successful and efficient. In that analysis, we always combined results from five different values of Δ*E*, and showed that accurate predictions of the rate could be obtained even with only a small number of simulations per set. We also wanted to investigate what the minimum number of different Δ*E* values we could use is, and also whether it was required to have the lowest Δ*E* values (which require the most simulation time to observe transitions). Figure 5 shows the result of this analysis, where for the worst CV, *R*_ee_, we systematically eliminate either 0, 1, 2, or 3 of our data sets, while maintaining a constant number of simulations within each data set. As was the case in Figure 4, we see that the estimate from the OPES-flooding estimator is systematically wrong in all cases. Interestingly, as seen in the top panel, adding more data sets with higher Δ*E* makes the OPESf estimate worse; in the lower panel, we see the first point using only the highest two Δ*E* is particularly bad, but adding more data at lower barriers slightly improves the estimate. In stark contrast, using the same data the EATRf estimator is relatively accurate when using just the lowest 2 barriers, and becomes systematically better as more data is added. It is also 2 log units better than the OPESf estimate with even just the highest two Δ*E* sets included, albeit with a large variance.

**Figure 5:**
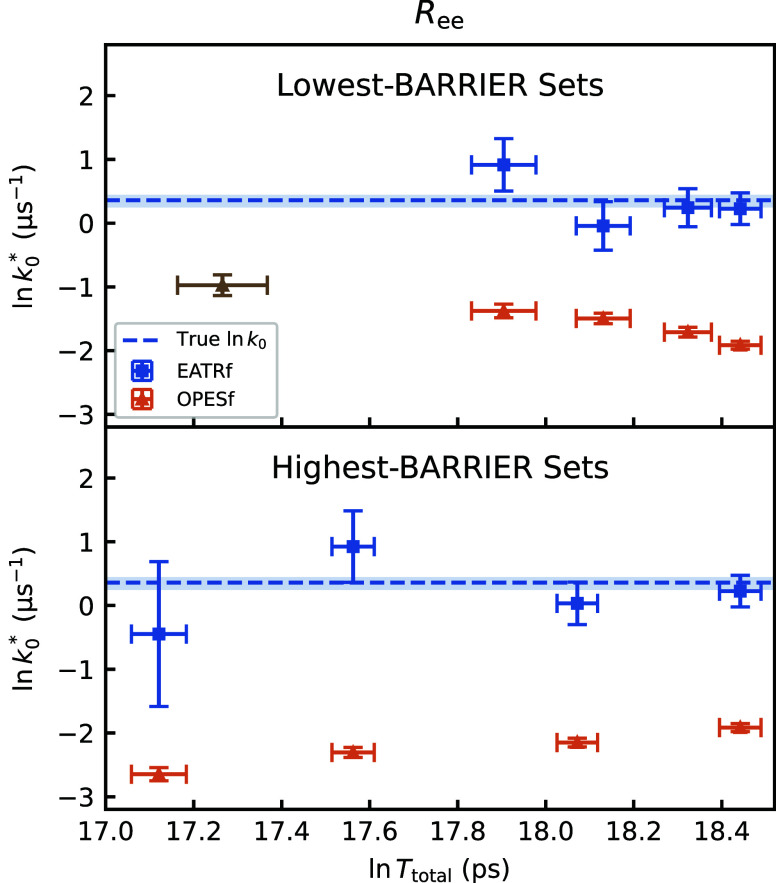
Predicted value of ln *k*_0_ from EATR-flooding and OPES-flooding on the *R*_ee_ CV with the lowest (top) and highest (bottom) values for Δ*E* with 100 simulations per set. The blue dashed line represents the rate obtained from unbiased simulations. Error bars indicate the standard error in bootstrap analysis.

Given that EATRf was conceived in the context of quasi-static biasing, we also performed simulations where we added a truly static Gaussian bias rather than using OPES. We performed otherwise identical simulations and analysis, adding a constant external potential to either *R*_ee_ or *Q*, described in Section S1.1. Figure S3 shows the EATRf analysis for these data, which shows equally or more accurate results as compared to the OPES data, despite the naive way in which the bias was constructed (Table 1).

Finally, to further demonstrate the generality of EATRf, we applied our workflow to data generated by iMetaD for ref 26, where the different forcing amounts are controlled by the frequency of hill deposition alone. As described in Section S1.2 and seen in Figure S4 and Table 1, this approach is as accurate as our EATRf results using OPES.

### 4.2 Cavity-Ligand Model

Although EATRf seems promising on a toy model, we want to ensure that it works for a larger and fully atomistic system. A persistent difficulty is finding a challenging benchmark system that still admits a known “ground truth” and which also has multiple possible choices of CVs to bias. For this study, we adopted a simple model of receptor–ligand unbinding that was originally developed to study the effect of collective dewetting on ligand dissociation.^34^ This system consists of a buckyball in a hydrophobic cavity, and it is known that the typical unbinding reaction occurs with the buckyball sliding sideways in the pocket before escaping, which allows the pocket to become hydrated, rather than coming directly out which creates a temporary vacuum.^35^ We have previously used this model to study the effect of mechanical pulling forces on the unbinding rates using iMetaD.^36^ However, we do not know the “true” unbinding rate, and an alternative approach, Markov state modeling, gives an estimate orders of magnitude faster than that from iMetaD.^37^ For this study, we therefore decided to modify the parameters of this model to produce a series of cavity-ligand models with lower binding affinities, from which we can select a system that displays the same qualitative behavior but for which unbinding occurs fast enough to allow us to estimate an unbiased rate.

As detailed in the SI, we reduced the ϵ parameters of the nonbonded interactions between the receptor and ligand atoms. Three different models with decreasing attractive interactions were evaluated. We computed the unbinding PMF using the MetaD procedure from our previous study,^36^ with results shown in Figure S5. As ϵ is lowered slightly (Tables S1 and S2), we see a systematic decrease in the barrier to unbinding. The third model (ϵ_3_) tested has a barrier approximately 10 kcal/mol lower and we proceeded to assess whether we could approximate its unbinding rate constant with unbiased MD.^36^ At the same time, as shown in Figure S5a,b, the underlying free-energy landscape has the same shape as in the original model, meaning we still expect unbinding to follow the same type of nontrivial unbinding pathway.

To estimate an unbiased rate constant for our model, we performed 360 total microseconds of unbiased MD launched from 48 different sampled starting points. This produced only five transitions. Using these data, we made a maximum likelihood estimate for the unbiased log-rate of 2.67 (with *k*_0_ in ms^–1^), with a highest-density 95% credible interval of 1.62 to 3.44 determined according to Section 6.3.

We then performed sets of 48 OPES simulations each for a series of Δ*E* values from 3 to 6 kcal/mol. We biased two CVs: the axial (*z*) and radial ( $\rho =\sqrt{x^{2}+y^{2}}$ ) distances from the ligand to the center of the cavity. For larger and more complex systems like this, we first examined the change in the logarithm of the rate constant with the logarithm of the average acceleration factor, which is presented in Figure S6. In practice, we have observed that when the system is not overbiased, this curve will be linear with a slope approximately equal to γ (equivalent to taking γ outside the average in eq 8). For very large Δ*E*, we observe that the points start to curve down, and we take this as a heuristic of which points to exclude for our EATRf analysis, as these likely correspond to overbiasing regimes.

Taking six values of Δ*E* (3, 3.5, 4, 4.5, 5, and 6 kcal/mol) we performed the EATRf analysis with results shown in Figure 6. When biasing the *z*-coordinate, we obtained 3.47 ± 0.34 as the estimate for ln *k*_0_ with *k*_0_ in ms^–1^ and γ = 0.93 ± 0.04. When biasing the ρ-coordinate, we obtained ln *k*_0_ = 3.50 ± 0.36 and γ = 0.88 ± 0.04. We find that *z*, the natural unbinding coordinate, has a high γ and a predicted rate constant consistent within uncertainty of our unbiased estimate. For ρ, we get a slightly lower γ as expected since it does not directly drive the unbinding. Using the traditional OPESf analysis from just our lowest barrier data produced ln *k*_0_ = 2.89 ± 0.06 for *z* and ln *k*_0_ = 2.50 ± 0.07 for ρ.

**Figure 6:**
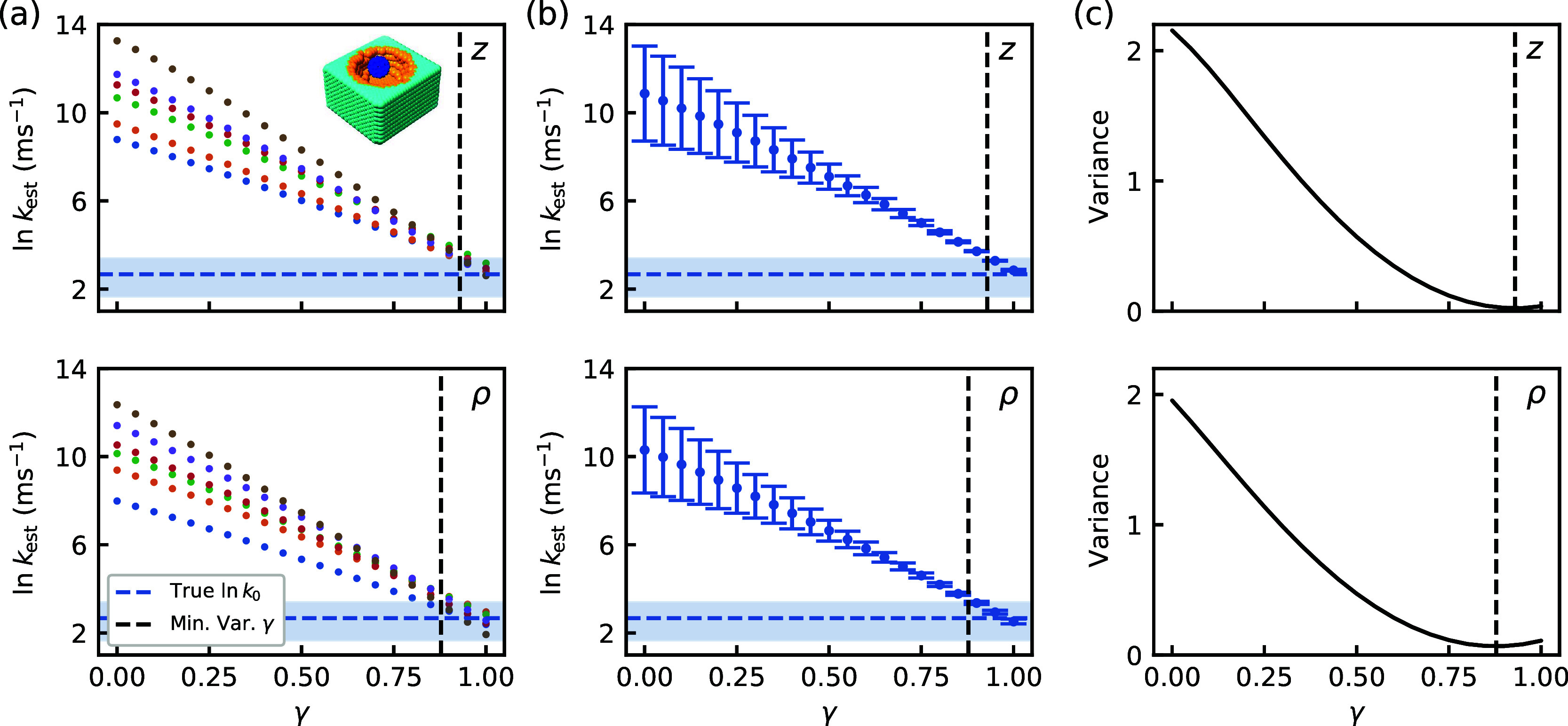
Results of EATR-flooding on the weakened cavity-ligand model. (a) Values of ln *k*_est_ predicted by eq 8 for each set of OPES simulations with each Δ*E* for various values of γ. (b) Value of ln *k*_est_ averaged over the sets for each value of γ. The variance of ln *k*_est_ across the sets is represented by error bars. (c) Variance in the predicted value of ln *k*_est_ across the sets of simulations. In (a,b), the horizontal dashed lines represent the estimate of ln *k*_0_ from unbiased MD and the 95% credible interval. In all panels, the vertical dashed lines represent the minimum variance value of γ.

Although water plays a crucial role in buckyball unbinding mechanisms,^35^ neither *z* nor ρ explicitly accounts for solvation effects. Despite this, we were surprised to find that both CVs yield high γ values, implying that they implicitly capture these solvation effects quite effectively in this case.

Although we do not have an estimate of the unbiased rate for the original “strong” model, we did perform EATR-flooding analysis as shown in Figure S7. In this case, we find that *z* alone is predicted to be a less ideal CV than in our weaker case (γ = 0.64) and this is not substantially improved by biasing *z* and ρ simultaneously (also γ = 0.64). The predicted unbinding times fall between those for MSM and previous iMetaD studies and are probably underestimates given our predicted difference in the barrier heights of the original and new models.

Returning to our weaker cavity model, because *z* and ρ were both found to be good CVs for biasing in this system and we have a wide estimate of the true rate, it is difficult to demonstrate the effect of EATRf. We therefore decided to define a set of CVs to demonstrate that EATRf still works even if the quality of the CV is worse than that of ρ. We first created a nuisance CV θ that represents the zenith angle of a chosen carbon atom (defined in PLUMED using a dihedral between that atom, the center of the buckyball, the center of the cavity, and an atom in the cavity that is aligned to the vertical axis), then we created four CVs, each of which is of the form ξ = *az* + *b*θ, where *a* ^2^ + *b* ^2^ = 1. We used *a* = 1.00, 0.95, 0.90, and 0.87. The results of performing EATRf and OPESf on these simulations are given in Figure 7. Adding weight to θ and removing weight from *z* in the CV is expected to make the CV worse for biasing, and in Figure 7, we can clearly see the effect of systematically making our biased CV worse, as the slope decreases and deviates from 1, which is the OPESf approximation.

**Figure 7:**
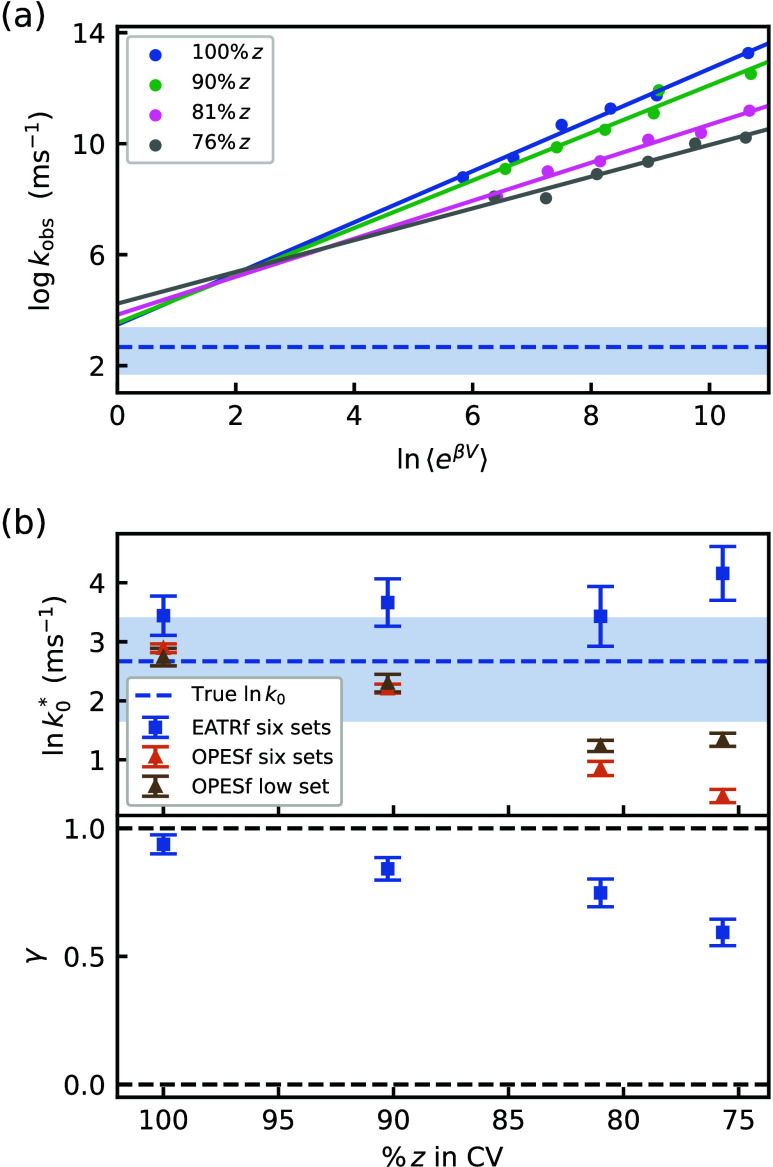
(a) Observed value of the log rate in the simulation sets while biasing a CV defined as linear combinations of *z* and θ, plotted against ln ⟨*e* ^βV^⟩, which acts as a measure of the amount of bias added. For worse CVs that have a larger contribution from θ, adding the same amount of bias has less of an effect on the observed rate. Solid lines are linear fits to the data, with slopes from top to bottom of 0.94, 0.84, 0.75, and 0.59. (b) The predicted value of ln *k*_0_ (top) and γ (bottom) from EATR-flooding and OPESf while biasing each of the CVs. Both the results of applying OPESf to all simulation sets and the results of applying OPESf only to the set with Δ*E* = 3.5 kcal/mol are shown. The *x*-axis is the value of *a* ^2^ where *a* is the coefficient of *z* in the linear combination. Mean γ values are 0.92, 0.86, 0.68, and 0.57, quite similar to the values of slope in (a), in accordance with the hypothesis in ref 26.

Performing our full EATRf analysis, we find that γ decreases systematically along with the fraction of θ introduced in Figure 7b. At the same time, this leads to a decrease in the OPESf rate estimate, i.e., a severe overestimate of the binding lifetime. In contrast, we were pleased to see that the rate prediction from EATRf is roughly the same even as the CV gets substantially worse.

## 5 Conclusions

In this study, we developed a variant of the EATR method that allows for a more general way of obtaining accurate rate constants when applying biasing to CVs of nonideal quality, including methods that apply a quasi-static bias where our previous formulation failed. The primary innovation is to run many sets of biased simulations, each with a different amount of bias. These multiple sets of biased data allow us to visualize whether the bias coordinate is efficient. Moreover, we can use the relationship between the amount of bias, the observed rate, and the unbiased rate to estimate the unbiased rate for each set of simulations as a function of the γ parameter. The value of γ that minimizes the variance in these estimates is taken as the γ estimate, and the average of the corresponding rate estimates at that γ is taken as the unbiased rate estimate for the method. We found that we can use multiple sets of simulations to measure the sensitivity of the observed rate on the bias, which determines the γ parameter for EATR.

EATR-flooding successfully reduces the complexity of our rate estimation approach by yielding a single γ parameter, but we note that this quality measure remains intrinsically tied to the specific biasing protocol employed. Our results in Table 1 show that while different methods—such as a fixed bias, iMetaD, or OPES—produce equally accurate rate constants for the same collective variables, their resulting γ values vary. This protocol dependence might persist even within a single framework; for instance, variations in OPES parameters, such as decoupling the bias factor from Δ*E*, could influence the metric. It is left for future work to theoretically understand the physical significance of γ and relate it to variations in biasing protocols.

For the cases we have evaluated, EATR-flooding produces consistent and accurate rate constant estimates when varying the choice of biased CV or CVs. Although this requires performing multiple sets of simulations, reasonable rates can be obtained with relatively few simulations per set and a small number of sets, such that there is only a relatively small increase in cost for a potentially substantial gain in accuracy. At first glance, EATR-flooding might appear more computationally expensive because it requires running multiple simulations at different barrier values. However, as shown in Figures 4 and 5, it can yield more accurate rate estimates for the same total computational cost. For instance, instead of allocating 60 simulations to the lowest affordable barrier, a practitioner could distribute the same computational effort across several batches (for example, 30 simulations at the lowest barrier and 30 at several higher barriers, which finish faster). This approach not only provides insight into the quality of the CV but also, in principle, yields a more accurate prediction. Conversely, if one only has a few simulations at high barriers, then EATR-flooding is indeed more expensive. However, relying solely on high-barrier simulations represents the least accurate baseline, where rate constants can be off by orders of magnitude. By investing additional simulations at lower barriers, the practitioner simultaneously verifies the quality of their chosen CV and obtains a significantly better rate estimate. Moreover, it was also shown that when enough bias has been added to reach the overbiasing regime, the rate becomes less sensitive to the further addition of bias, allowing us to diagnose when the system is being truly overbiased in a manner that may prevent extracting a true rate.

By extending the EATR method to work with OPES simulations (or related methods such as VES), we have not only improved the capability for rapid biasing, but we have also opened up the possibility to gain control over the biasing with OPES, using its built-in limit on maximum bias, Δ*E*, and optionally the use of an excluded region. This will allow us to tackle kinetics computations for challenging biomolecular problems, such as in our previous work evaluating the force-dependence of the unbinding of the protein vinculin from actin, where OPESf seemed to provide more stable estimates of kinetics than iMetaD.^38^ We would now like to revisit those calculations to determine whether additional OPES simulations varying Δ*E* will substantially change our estimates of vinculin binding lifetimes, and whether this approach can allow us to find a better set of CVs for efficient lifetime computations.

As described in the theory section, we are not limited to combining sets of OPES simulations; in principle EATRf should apply to other biasing methods as well, including time-dependent approaches like MetaD. While our preliminary results in Figure S4 show that EATRf can be applied directly to iMetaD data using, as an example, different hill addition rates, further work is required to determine whether optimal choices for iMetaD parameters are required. Moreover, we would like to investigate whether we can combine our two approaches to take advantage of both the increase of bias between sets of simulations and the increase of bias over the course of each simulation to determine the sensitivity of the rate to bias, and thus obtain a more accurate estimate of γ.

## 6 Computational Methods

MD simulations were performed in LAMMPS^39^ and GROMACS^40^ using standard protocols described below. Enhanced sampling simulations using both MetaD and OPES were performed with the PLUMED open source sampling library.^28^ We have previously contributed an example of computing rates of such systems under applied mechanical force in the PLUMED-tutorials resource.^41^

### 6.1 MD Simulation Details

The molecular dynamics simulations of the Go̅-like model of protein G were performed using the 23 Jun 2022 version of the LAMMPS^39^ software package patched with PLUMED 2.8.3.^28^ The OPES simulation data where the *Q* and *R*_ee_ CVs were used for biasing were the same data as used in the Supporting Information of ref 26. The data where the *R*_g_ and *R*_g_/*R*_ee_ CVs were used for biasing were collected using similar scripts. The simulations were run with a 10 fs time step, using the Nosé-Hoover chain thermostat at 312 K with a damping parameter of 1 ps and a chain length of 3. Each simulation was stopped once the protein unfolded, which we defined to occur once the *Q* CV dropped below 0.35.

The cavity-ligand model was adapted from our previous setup in ref 36, which was the same as that in refs 34, 35, 37. The model is composed of a cube of hexagonally close-packed carbonlike atoms with an ellipsoidal cavity and a buckyball (C60) ligand, solvated with TIP4P water molecules. The cavity and ligand atoms have attractive nonbonded interactions. The model used in this project was adjusted to weaken these interactions, as described in the SI.

The MD simulations for the cavity-ligand model were performed using GROMACS 2023.2 ^40^ patched with PLUMED 2.9.^28^ The simulations were run in a periodic box with a 2 fs time step at 300 K, using the V-Rescale temperature coupling. Each simulation ended once the ligand unbound from the cavity or 500 ns had been simulated. Ligand unbinding was defined to occur once the *z*-distance increased past 15 Å.

### 6.2 EATR-Flooding

The rate estimations were performed on collections of OPES simulation sets, where each simulation set was run with a different value for Δ*E*, the BARRIER parameter in PLUMED. The protein G simulations were run with Δ*E* ranging from 5 to 15 kJ/mol with 2 kJ/mol increments, of which we show results for the bottom five sets. The excluded regions for the bias in the protein G simulations for each CV were *Q* < 0.65, *R*_g_ > 1.15 nm, and *R*_ee_ > 2.9 nm. For the 2D *R*_g_, *R*_ee_ CV, bias was excluded from the union of the excluded regions for the separate CVs. The cavity-ligand model simulations shown in the main text were run with Δ*E* ranging from 3 to 6 kcal/mol in 1 kcal/mol increments, plus 3.5 and 4.5 kcal/mol. Those in the SI for the original model from ref 34 used Δ*E* from 13 to 16 kcal/mol in 1 kcal/mol increments, plus 13.5 and 14.5 kcal/mol. We did not find that adding an excluded region substantially improved results, while incurring some extra cost (see Figure S8 and Table S3), hence no excluded region was used for the cavity model calculations in the main text.

While analyzing the protein G simulation sets, the biased rate constants were determined by fitting the final transition times to a Poisson distribution. The same was done for the original cavity-ligand model. However, for the new cavity-ligand model, we instead used maximum likelihood estimation to determine the biased rate constants because some simulations were stopped prematurely, which would influence the observed rate constant.

The ensemble average ⟨*e* ^*βγV*^⟩ for a given value for Δ*E* was estimated by first averaging the quantity *e* ^*βγV*(*t*)^ across all running simulations in the set at time *t*, then averaging the result over time. This ordering was done to be consistent with our earlier EATR approach, but we also tested doing the calculation in the other order with minor quantitative differences. We applied eq 8 to each simulation set to get a set of ln *k*_0_ estimates as a function of γ, then minimized the variance in the ln *k*_0_ estimates using the bounded version of Brent’s algorithm implemented in the SciPy Python package.^42^ The minimizing value of γ and the mean of the corresponding ln *k*_0_ values are reported as the γ and ln *k*_0_ estimates in this method.

### 6.3 Error Analysis

All error values, unless otherwise stated, were obtained by bootstrapping.^43^ In this method, we calculate the error in some statistic from a number of data sets constructed by randomly sampling the original data set with replacement. We resampled 1000 times and calculated the relevant quantity for each resampling (usually the log-rate or γ), then took the standard deviation across the resamplings as the error in that quantity. The error in the unbiased unfolding rate of the protein G model is a 95% confidence interval obtained using the bootstrapping method implemented in SciPy.^42^

For the estimation of the unbiased rate for the weakly bound cavity model, we used Bayesian analysis to estimate a 95% credible interval from our set of simulations where we observed five transitions within 360 μs. The posterior distribution, π­(*k*_0_|*N*, *T*_total_), given a number of transitions *N* and a total simulation time *T*_total_ was determined following ref 44. using the noninformative prior distribution π­(*k*_0_) ∝ 1/*k*_0_, leading to

10 $$\pi \left(k_{0}|N,T_{\mathrm{total}}\right)\propto k_{0}^{N-1}\exp\left(-k_{0}T_{\mathrm{total}}\right)$$

This was then converted to the posterior distribution for ln *k*_0_. The highest-density credible interval is obtained from this posterior distribution by finding two ln *k*_0_-values *a* and *b* where *P*(*a* < ln *k*_0_ < *b*|*N*, *T*_total_) = 95% and π­(ln *k*_0_ = *a*|*N*, *T*_total_) = π­(ln *k*_0_ = *b*|*N*, *T*_total_).

## Supplementary Material



## Data Availability

Data and analysis scripts are available from the GitHub repository for this paper: https://github.com/hocky-research-group/EATRf-paper-2026. The general EATR analysis framework software is available from https://github.com/hocky-research-group/EATR-rate-analysis.

## Notes

Additional details of EATRf analysis for the protein G system including results using a static bias, and results using iMetaD. Data on parametrizing the cavity-ligand system used in the study are provided, as well as EATRf results using an excluded region and results on the original “strong” cavity model (PDF)

## References

[1] Ferruz N., De Fabritiis G. (2016). Binding kinetics
in drug discovery. Mol. Inf..

[2] Lotz S. D., Dickson A. (2018). Unbiased molecular
dynamics of 11 min timescale drug
unbinding reveals transition state stabilizing interactions. J. Am. Chem. Soc..

[3] Bernetti M., Masetti M., Rocchia W., Cavalli A. (2019). Kinetics of drug binding
and residence time. Annu. Rev. Phys. Chem..

[4] Nunes-Alves A., Kokh D. B., Wade R. C. (2020). Recent
progress in molecular simulation
methods for drug binding kinetics. Curr. Opin.
Struct. Biol..

[5] Ahn S.-H., Ojha A. A., Amaro R. E., McCammon J. A. (2021). Gaussian-accelerated
molecular dynamics with the weighted ensemble method: A hybrid method
improves thermodynamic and kinetic sampling. J. Chem. Theory Comput..

[6] Smith Z., Branduardi D., Lupyan D., D’Arrigo G., Tiwary P., Wang L., Krilov G. (2025). Toward automated physics-based
absolute drug residence time predictions. J.
Chem. Inf. Model..

[7] Karplus M., McCammon J. A. (2002). Molecular dynamics simulations of
biomolecules. Nat. Struct. Biol..

[8] Schlick T., Portillo-Ledesma S., Myers C. G., Beljak L., Chen J., Dakhel S., Darling D., Ghosh S., Hall J., Jan M., Liang E., Saju S., Vohr M., Wu C., Xu Y., Xue E. (2021). Biomolecular Modeling and Simulation: A Prospering
Multidisciplinary Field. Annu. Rev. Biophys..

[9] Tuckerman, M. E. Statistical Mechanics: Theory and Molecular Simulation; Oxford University Press, 2023.

[10] Hénin J., Lelièvre T., Shirts M., Valsson O., Delemotte L. (2022). Enhanced Sampling
Methods for Molecular Dynamics Simulations. Living J. Comput. Mol. Sci..

[11] Kästner J. (2011). Umbrella sampling. Wiley Interdiscip. Rev. Comput. Mol. Sci..

[12] Laio A., Parrinello M. (2002). Escaping free-energy
minima. Proc. Natl. Acad. Sci. U.S.A..

[13] Barducci A., Bussi G., Parrinello M. (2008). Well-tempered
metadynamics: a smoothly
converging and tunable free-energy method. Phys.
Rev. Lett..

[14] Invernizzi M., Parrinello M. (2020). Rethinking metadynamics: From bias potentials to probability
distributions. J. Phys. Chem. Lett..

[15] Valsson O., Parrinello M. (2014). Variational
approach to enhanced sampling and free
energy calculations. Phys. Rev. Lett..

[16] Bussi G., Laio A. (2020). Using metadynamics
to explore complex free-energy landscapes. Nat.
Rev. Phys..

[17] Voter A. F. (1997). Hyperdynamics:
Accelerated molecular dynamics of infrequent events. Phys. Rev. Lett..

[18] Tiwary P., Parrinello M. (2013). From Metadynamics to Dynamics. Phys. Rev. Lett..

[19] Salvalaglio M., Tiwary P., Parrinello M. (2014). Assessing
the Reliability of the
Dynamics Reconstructed from Metadynamics. J.
Chem. Theory Comput..

[20] McCarty J., Valsson O., Tiwary P., Parrinello M. (2015). Variationally
optimized free-energy flooding for rate calculation. Phys. Rev. Lett..

[21] Debnath J., Parrinello M. (2020). Gaussian mixture-based
enhanced sampling for statics
and dynamics. J. Phys. Chem. Lett..

[22] Ray D., Ansari N., Rizzi V., Invernizzi M., Parrinello M. (2022). Rare event kinetics from adaptive
bias enhanced sampling. J. Chem. Theory Comput..

[23] Ray D., Parrinello M. (2023). Kinetics from
Metadynamics: Principles, Applications,
and Outlook. J. Chem. Theory Comput..

[24] Blumer O., Reuveni S., Hirshberg B. (2024). Short-Time
Infrequent Metadynamics
for Improved Kinetics Inference. J. Chem. Theory
Comput..

[25] Palacio-Rodriguez K., Vroylandt H., Stelzl L. S., Pietrucci F., Hummer G., Cossio P. (2022). Transition Rates and Efficiency of
Collective Variables from Time-Dependent Biased Simulations. J. Phys. Chem. Lett..

[26] Mazzaferro N., Sasmal S., Cossio P., Hocky G. M. (2024). Good Rates
From
Bad Coordinates: The Exponential Average Time-dependent Rate Approach. J. Chem. Theory Comput..

[27] Grubmüller H. (1995). Predicting
slow structural transitions in macromolecular systems: Conformational
flooding. Phys. Rev. E.

[28] Tribello G. A., Bonomi M., Branduardi D., Camilloni C., Bussi G. (2014). PLUMED 2: New feathers for an old
bird. Comput.
Phys. Commun..

[29] Bonomi M., Bussi G., Camilloni C., Tribello G. A., Banáš P., Barducci A., Bernetti M., Bolhuis P. G., Bottaro S., Branduardi D. (2019). Promoting transparency and reproducibility
in enhanced molecular simulations. Nat. Methods.

[30] Voter A. F. (1997). A method
for accelerating the molecular dynamics simulation of infrequent events. J. Chem. Phys..

[31] Dudko O. K., Graham T. G. W., Best R. B. (2011). Locating the Barrier for Folding
of Single Molecules under an External Force. Phys. Rev. Lett..

[32] Graham T. G. W., Best R. B. (2011). Force-Induced Change
in Protein Unfolding Mechanism:
Discrete or Continuous Switch?. J. Phys. Chem.
B.

[33] Mazzaferro N., Lee S., Cossio P., Tiwary P., Hocky G. M. (2025). Using Time Dependent
Rate Analysis to Evaluate the Quality of Machine Learned Reaction
Coordinates for Biasing and Computing Kinetics. J. Phys. Chem. B.

[34] Mondal J., Morrone J. A., Berne B. J. (2013). How hydrophobic
drying forces impact
the kinetics of molecular recognition. Proc.
Natl. Acad. Sci. U.S.A..

[35] Tiwary P., Mondal J., Morrone J. A., Berne B. J. (2015). Role of water and
steric constraints in the kinetics of cavity–ligand unbinding. Proc. Natl. Acad. Sci. U.S.A..

[36] Peña
Ccoa W. J., Hocky G. M. (2022). Assessing models of force-dependent
unbinding rates via infrequent metadynamics. J. Chem. Phys..

[37] Ahalawat N., Bandyopadhyay S., Mondal J. (2020). On the role of solvent in hydrophobic
cavity–ligand recognition kinetics. J.
Chem. Phys..

[38] Peña
Ccoa W. J., Mukadum F., Ramon A., Stirnemann G., Hocky G. M. (2025). A direct computational assessment of vinculin-actin
unbinding kinetics reveals catch–bonding behavior. Proc. Natl. Acad. Sci. U.S.A..

[39] Thompson A. P., Aktulga H. M., Berger R., Bolintineanu D. S., Brown W. M., Crozier P. S., In’t Veld P. J., Kohlmeyer A., Moore S. G., Nguyen T. D. (2022). LAMMPS-a
flexible simulation tool for particle-based materials modeling at
the atomic, meso, and continuum scales. Comput.
Phys. Commun..

[40] Abraham M. J., Murtola T., Schulz R., Páll S., Smith J. C., Hess B., Lindahl E. (2015). GROMACS: High performance
molecular simulations through multi-level parallelism from laptops
to supercomputers. SoftwareX.

[41] Tribello G. A., Bonomi M., Bussi G., Camilloni C., Armstrong B. I., Arsiccio A., Aureli S., Ballabio F., Bernetti M., Bonati L. (2025). PLUMED
Tutorials: A
collaborative, community-driven learning ecosystem. J. Chem. Phys..

[42] Virtanen P., Gommers R., Oliphant T. E., Haberland M., Reddy T., Cournapeau D., Burovski E., Peterson P., Weckesser W., Bright J., van der Walt S. J., Brett M., Wilson J., Millman K. J., Mayorov N., Nelson A. R. J., Jones E., Kern R., Larson E., Carey C. J., Polat İ., Feng Y., Moore E. W., VanderPlas J., Laxalde D., Perktold J., Cimrman R., Henriksen I., Quintero E. A., Harris C. R., Archibald A. M., Ribeiro A. H., Pedregosa F., van Mulbregt P. (2020). SciPy 1.0
Contributors, SciPy 1.0: Fundamental Algorithms for Scientific Computing
in Python. Nat. Methods.

[43] Efron B. (1979). Another Look
at the Jackknife. Ann. Stat..

[44] Ibrahim, J. G. ; Chen, M.-H. ; Sinha, D. Bayesian Survival Analysis; Springer Science & Business Media, 2001.

