# Supplementary material for: Stepping Up Enhanced Rate Calculations with EATR-Flooding
Source: J Chem Theory Comput. 2026 Jul 29;22(15):7570–80. doi: 10.1021/acs.jctc.6c00883 (PMC13471737; doi:10.1021/acs.jctc.6c00883)
Supplement: Supplementary file 1 [file ct6c00883_si_001.pdf]

Supporting Information for: Stepping up enhanced rate calculations with EATR-flooding

Nicodemo Mazzaferro,[†] Willmor J. Peña Ccoa,[†] Pilar Cossio,[*,‡,¶] and Glen M. Hocky[*,†,§]

†*Department of Chemistry, New York University, NY, 10003, USA*

‡*Center for Computational Mathematics, Flatiron Institute, New York, 10010, USA*

¶*Center for Computational Biology, Flatiron Institute, New York, 10010, USA*

§*Simons Center for Computational Physical Chemistry, New York University, NY, 10003, USA*

E-mail: pcossio@flatironinstitute.org; hockyg@nyu.edu

1 Further results for protein G

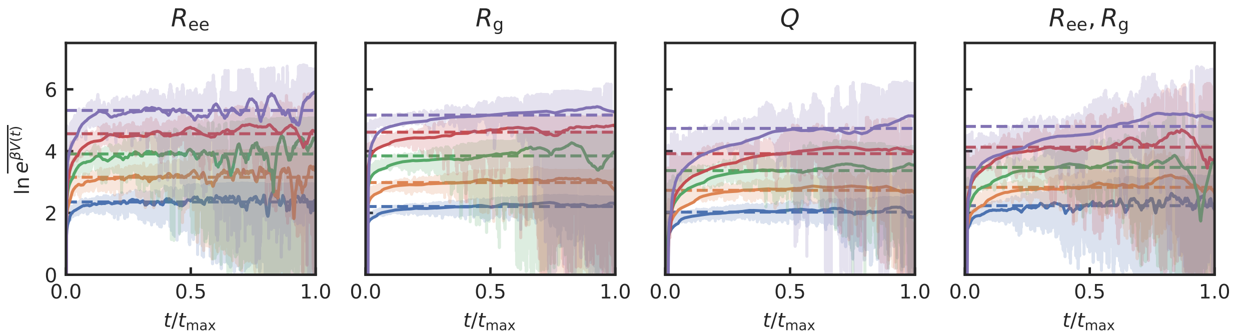

Figure S1: The log of the value of $e^{\beta V(t)}$ averaged over all simulations in each set (low opacity). The solid lines represent the log of the time average of $\overline{e^{\beta V(t)}}$ over a window of 25000 steps, and the horizontal dashed lines represent the time average from 0 up to the maximum transition time, which approximates the log of the ensemble average $\ln \left\langle e^{\beta V} \right\rangle$ in the folded state.

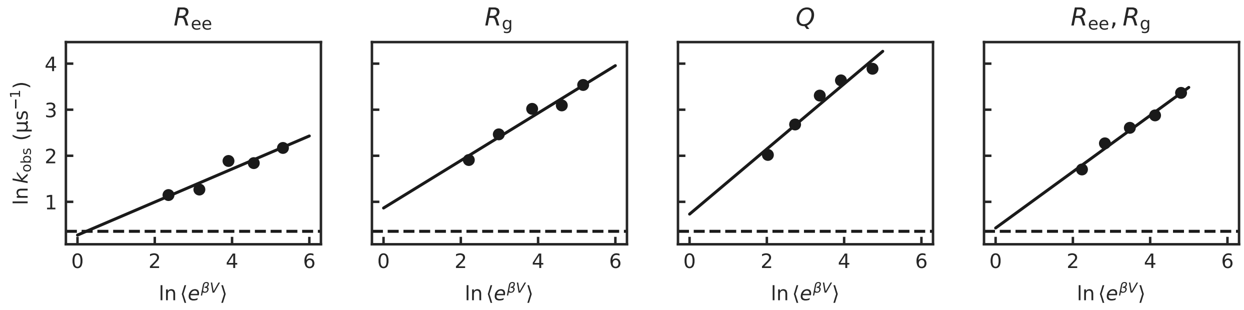

Figure S2: The logarithm of the observed rate plotted against $\ln \left\langle e^{\beta V} \right\rangle$ in the folded state of the protein G model, estimated as the log of the time average of $\overline{e^{\beta V(t)}}$. The horizontal dashed line represents the value of $\ln k_0$. Increasing $\ln \left\langle e^{\beta V} \right\rangle$ for poor CVs has less of an effect on $\ln k_{obs}$ than for good CVs. The EATR-slope approach takes the y-intercept of the best fit line to be the estimate for $\ln k_0$ and the slope to be the estimate for γ.

1.1 Static biasing

Because the EATR-flooding theory requires the bias to be effectively static, we decided to also run the analysis on a collection of simulation sets with exactly static biasing potentials. We ran six sets of simulations each for the Q and R_{ee} CVs of the protein G system, each with nominal bias levels of 4, 5, 6, 7, 8, and 10 kJ/mol.

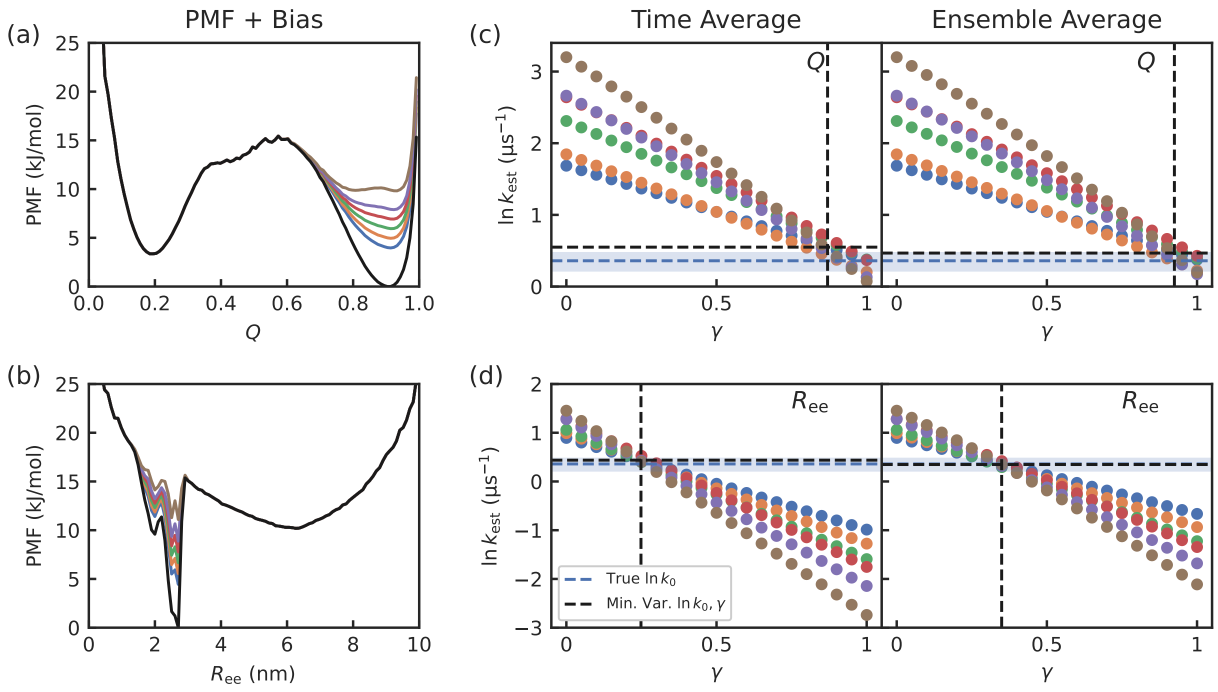

Figure S3: (a), (b) The PMFs of the protein G model for Q and R_{ee}. The unbiased PMF is shown in black while the biased PMF for each bias level is colored. (c), (d) The values of $\ln k_0$ predicted by Eq. 8 for each set of simulations for various values of γ, by approximating the expected value $\langle e^{\beta\gamma V}\rangle$ using the time average of the simulation average (left) and the directly estimated ensemble average (right).

For the Q CV, we applied a bias potential of the form

$$V(Q) = H \exp\left(-(Q-\mu)^2/a^2\right) ,$$

where H is the nominal bias level. We used $\mu = 0.9$ and $a = 0.13$.

Using the same EATR-flooding procedure as in the main text, we obtain $\ln k_0 = 0.53 \pm 0.16$ and $\gamma = 0.88 \pm 0.07$. The biased PMFs and the values of $\ln k_0$ obtained from Eq. 8 are given in Fig. S3a,c.

Because we have the exact expression for the bias, as well as a good estimate for the potential of mean force (PMF) in the folded state, we decided to also directly calculate the $\ln\langle e^{\beta\gamma V}\rangle$ term in Eq. 8 using

$$\langle A\rangle \approx \frac{\sum_i A(\xi_i) e^{-\beta(F(\xi_i)+V(\xi_i))}}{\sum_i e^{-\beta(F(\xi_i)+V(\xi_i))}} ,$$

where ξ_i are evenly spaced values for the CV ξ in the reactant state and $F(\xi_i)$ is the PMF of ξ at ξ_i. Using this instead yields $\ln k_0 = 0.48 \pm 0.14$ and $\gamma = 0.92 \pm 0.06$.

For the R_{ee} CV, we applied a bias potential of the form

$$V(R_{\mathrm{ee}}) = H\left(0.9 \exp\left(-(R_{\mathrm{ee}}-\mu_1)^2/a_1^2\right) + \exp\left(-(R_{\mathrm{ee}}-\mu_2)^2/a_2^2\right) + 0.5 \exp\left(-(R_{\mathrm{ee}}-\mu_3)^2/a_3^2\right)\right) ,$$

where we used $\mu_1 = 2.45$ nm, $\mu_2 = 2.68$ nm, $\mu_3 = 1.9$ nm, $a_1 = 0.2$ nm, $a_2 = 0.12$ nm, and $a_3 = 0.3$ nm.

Estimating $\ln\langle e^{\beta\gamma V}\rangle$ as in the main text results in $\ln k_0 = 0.40 \pm 0.18$ and $\gamma = 0.31 \pm 0.07$. Estimating it as above instead results in $\ln k_0 = 0.34 \pm 0.18$ and $\gamma = 0.35 \pm 0.07$. The biased PMFs and the values of $\ln k_0$ obtained from Eq. 8 are given in Fig. S3b,d.

1.2 Infrequent metadynamics biasing

We also decided to run the analysis on a set of iMetaD simulations to see if this procedure also works for non-static biasing potentials. We applied the EATR-flooding approach to the iMetaD

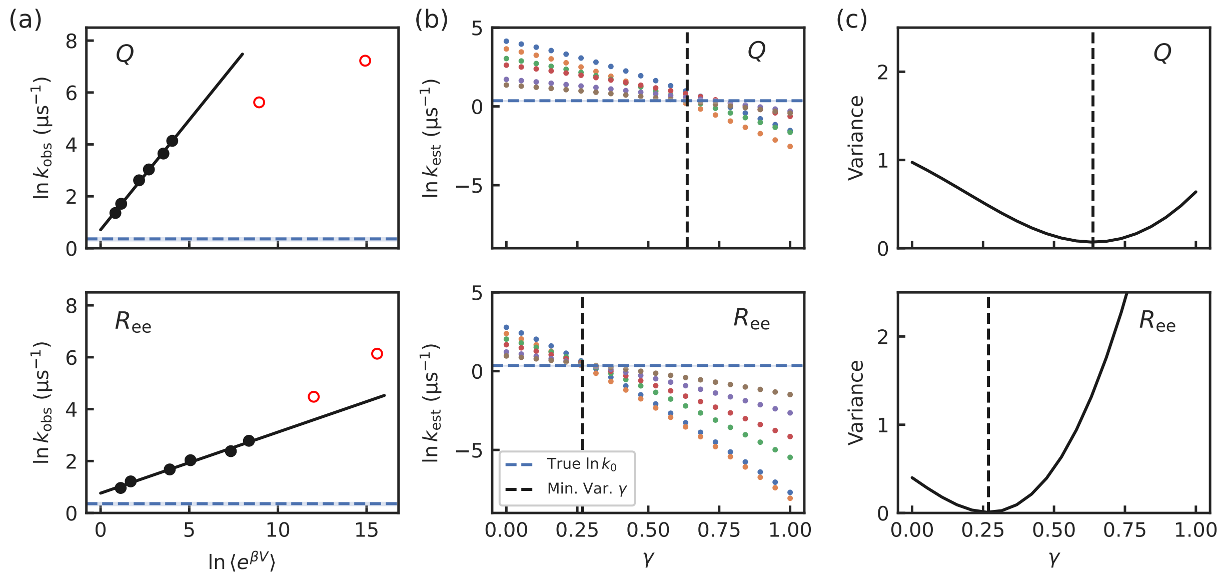

Figure S4: (a) The observed rate constant $\ln k_{\text{obs}}$ plotted against the $\ln\left\langle e^{\beta V}\right\rangle$ in the folded state of the protein G model, estimated as the average acceleration factor $\ln\overline{\alpha_i}$. The red open points are the sets of simulations which deviate from linearity, and are discarded. (b) The values of $\ln k_0$ predicted by Eq. 8 for each set of simulations for various values of γ (c) The variance in the predicted value of $\ln k_0$ across the sets of simulations. The horizontal dashed lines represent the true value of $\ln k_0$, with the 95% confidence interval represented by the blue shaded region. The vertical dashed lines represent the minimum variance value of γ.

simulations of the protein G model which were run with different bias deposition paces and were analyzed in Ref. 26. The simulations were run using well-tempered MetaD using 10.0 for the BIASFACTOR parameter, with a hill height of 0.4 kJ/mol and σ of 0.06 nm for R_{ee}, and a hill height of 0.6 kJ/mol and σ of 0.02 for Q. Each set of simulations was run using a different PACE parameter, from 1 ps to 10 ns per hill deposition.

First, the linear fit of $\ln k_{\mathrm{obs}}$ as a function of $\ln \left\langle e^{\beta V} \right\rangle$ was assessed as shown in Fig. S4a. The value of $\ln k_{\mathrm{obs}}$ was obtained by taking the reciprocal of the mean transition time, as the transition time distribution was expected to be non-Poissonian and therefore fitting the CDF would not be appropriate. The value of $\ln \left\langle e^{\beta V} \right\rangle$ was estimated by taking the final value of the acceleration factor for each simulation

$$\alpha_i = \frac{1}{t_i} \int_0^{t_i} e^{\beta V(\xi(t'),t')}\, dt'$$

and averaging across all simulations in the set. The fastest paces corresponding to deposition times of 1 and 10 ps were observed to deviate from the expected linear behavior, and were thus discarded.

The EATR-flooding protocol, where $\ln \left\langle e^{\beta\gamma V} \right\rangle$ was estimated by taking the simulation average before the time average, was then applied to the remaining data, as shown in Fig. S4b,c. For Q, the least-variance value for γ was 0.67 ± 0.06 and the corresponding value for $\ln k_0^*$ was 0.47 ± 0.17 with k_0^* in (μs^{-1}). For R_{ee}, γ^* was 0.28 ± 0.01 and the corresponding value for $\ln k_0^*$ was 0.43 ± 0.06.

2 Further results for the cavity-ligand model

The receptor is a semi-hollow cube that has a cavity which is half an ellipsoid of radius ~11 Å. Moreover, the cube consists of two types of hydrophobic atoms; the cavity atoms (CP) and the wall atoms (CW) (the atoms in the cavity have a higher attraction to the ligand atoms (CF) than do the wall atoms), and the whole complex model is solvated with TIP4P water. Parameters for non-bonded interactions between receptor and ligand atoms were determined using GROMACS' combination rule 3, where $\sigma_{ij} = \sqrt{\sigma_i \sigma_j}$ and $\epsilon_{ij} = \sqrt{\epsilon_i \epsilon_j}$. The ϵ values were reduced while keeping

the ratio between ϵ_{CP-CF} and ϵ_{CW-CF} fixed.

Table S1: Lennard-Jones parameters for the receptor and ligand atoms. CW, CP, and CF refer to the wall, cavity, and fullerene atoms respectively.

atom-type	σ(nm)	$\epsilon(\frac{\text{kJ}}{\text{mol}})$
CF	0.3500	0.276144
CW	0.4152	0.002400
CP	0.4152	0.008000

Table S2: Lennard-Jones parameters for non-bonded interactions between receptor and ligand atoms. Parameter ϵ_1 was used in the original model, while ϵ_3 was used for the fast unbinding model. The non-bonded interactions of the CP-CP, CW-CP, and CW-CW pairs were excluded from the total energy by setting their LJ parameters to 0 and the entire lattice was position restrained with a 1000 $\frac{kJ}{mol\,nm^2}$ force constant.

i	j	σ(nm)	$\epsilon_1(\frac{\text{kJ}}{\text{mol}})$	$\epsilon_2(\frac{\text{kJ}}{\text{mol}})$	$\epsilon_3(\frac{\text{kJ}}{\text{mol}})$	$\epsilon_4(\frac{\text{kJ}}{\text{mol}})$
CF	CP	0.3812	0.04700	0.03600	0.02500	0.01850
CF	CW	0.3812	0.02574	0.01972	0.01369	0.01015
CW	CW	0.4152	0.00000	0.00000	0.00000	0.00000
CP	CP	0.4152	0.00000	0.00000	0.00000	0.00000
CP	CW	0.4152	0.00000	0.00000	0.00000	0.00000

In Fig. S5C,D, Topology 1 (top. 1) describes the original model and has the highest barrier to unbinding on the z CV. Topology 2 (top. 2) has a lower barrier but remains high and kinetics were still too slow. Topology 4 has the lowest barrier, but the timescale of the unbinding kinetics was reduced to less than a microsecond. Thus, the second-weakest model (top. 3) was chosen for benchmarking.

Using the WTMetaD approach, FES calculations were performed for all models, including the original, biasing both the axial distance, z, and radial distance, ρ. The biasing parameters were: HEIGHT=0.478, SIGMA=0.3,0.1, BIASFACTOR=10, and PACE=300 for the modified models. For the original model, only one parameter was different, BIASFACTOR=12, which was higher to obtain comparable sampling within 100 ns. Restraining potentials (PLUMED UPPER_WALLS) were applied for the axial and radial distance CVs at 21 Å and 12 Å, respectively, to limit sampling within the periodic box.

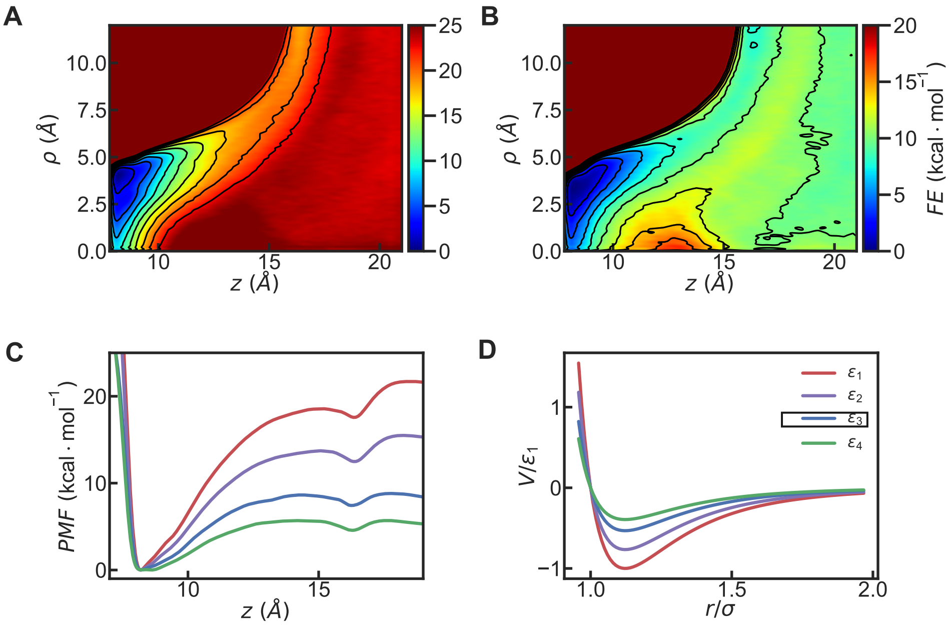

Figure S5: FES of original (A) and fast unbinding model, ϵ_3 (B), as a function of ρ and z. (C) Projection of FES onto axial distance, z, for all models. (D) LJ potential for interactions between cavity and ligand atoms (CP-CF) in original and modified models, where potential is scaled by ϵ_1 (see Table S2) and distance is scaled by σ.

3 Original cavity-ligand model results

For the original cavity-ligand model, we performed simulations with a set of ΔE values where we biased z alone, both z and ρ, and ρ alone. For all collections of simulation sets, we ran 6 sets of simulations with ΔE varying from 13 to 16 kcal/mol, except we did not run the 13 kcal/mol set for ρ alone. Because of a few outliers in the data set, the observed unbinding rates in the simulations could not be estimated using the maximum likelihood estimator. $\ln k_{\mathrm{obs}}$ was estimated by fitting the CDF instead, where in the case where a simulation ended early without transitioning, the CDF is fit only up to the time where that simulation ended. The results from EATRf are given in Fig. S7. The minimum-variance residence time we obtained from EATRf is $\tau_0 = 0.023$ s ($\gamma = 0.64$) when biasing z alone, $\tau_0 = 0.021$ s ($\gamma = 0.64$) when biasing z and ρ together, and $\tau_0 = 0.097$ s ($\gamma = 0.67$) when biasing ρ alone. This is between the residence time estimates from Markov state modeling ($\tau_0 = 0.00055$ s)[37] and previous infrequent metadynamics studies ($\tau_0 = 3863$ s, $\tau_0 = 2000$ s).[34,35]

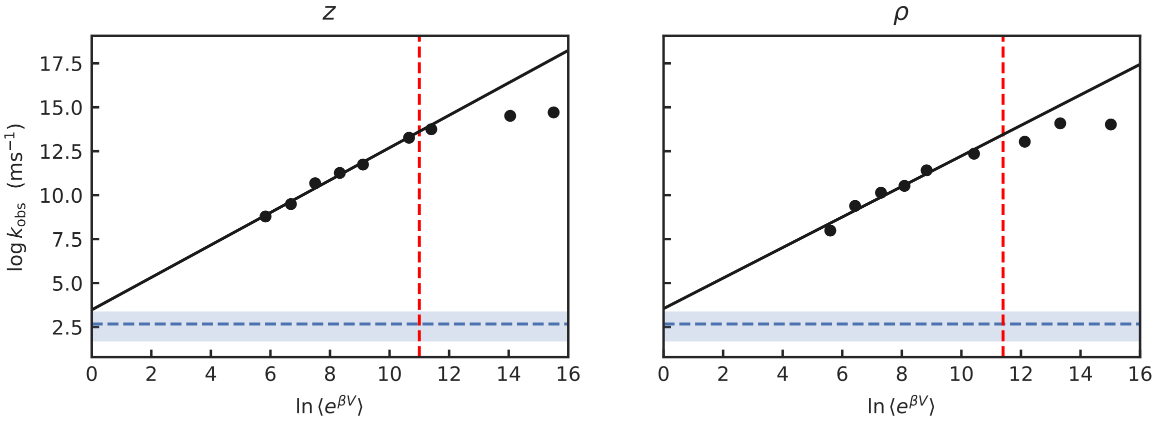

Figure S6: The logarithm of the observed rate plotted against $\ln\left\langle e^{\beta V}\right\rangle$ in the bound state of the cavity-ligand model, estimated as the log of the time average of $\overline{e^{\beta V(t)}}$. The horizontal dashed line represents the value of $\ln k_0$. Only points to the left of the red vertical dashed line were used in the EATRf analysis in the main text, as the rate becomes less sensitive to bias past that point, demonstrated by the deviation from linear behavior.

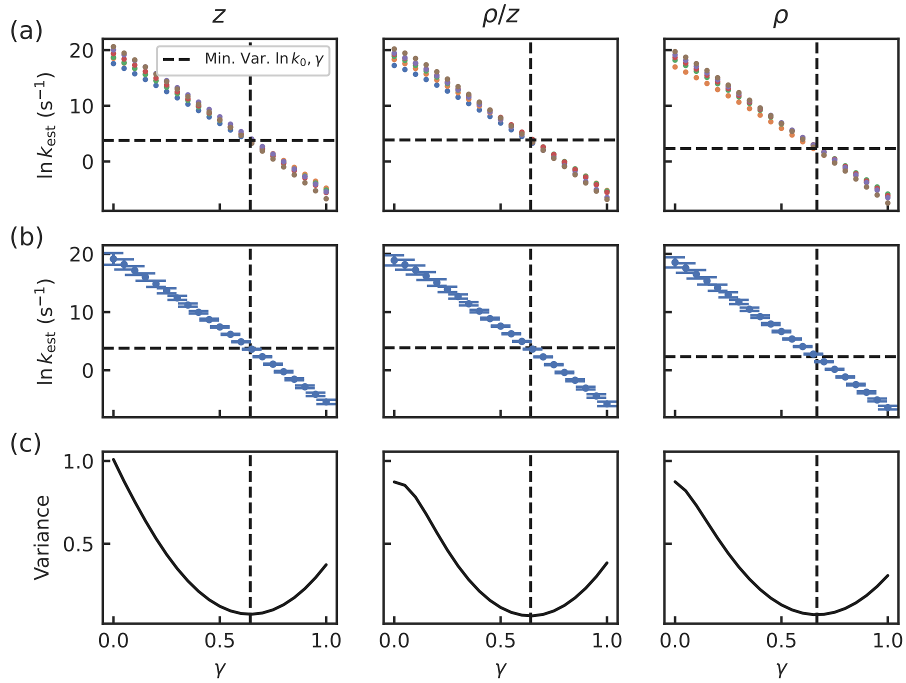

Figure S7: The results of EATRf on the original cavity-ligand model. (a) The values of $\ln k_0$ predicted by Eq. 8 for each set of OPES simulations with each BARRIER parameter for various values of γ. (b) The value of $\ln k_0$ averaged over the sets of simulations for each potential value of γ. The variance of $\ln k_0$ across the sets is represented by error bars. (c) The variance in the predicted value of $\ln k_0$ across the sets of simulations. The dashed lines represent the minimum variance value of γ, which is the value that causes all sets to give the same value of $\ln k_{\text{est}}$, and the corresponding $\ln k_0^*$.

4 Effect of excluded region on unbinding calculations

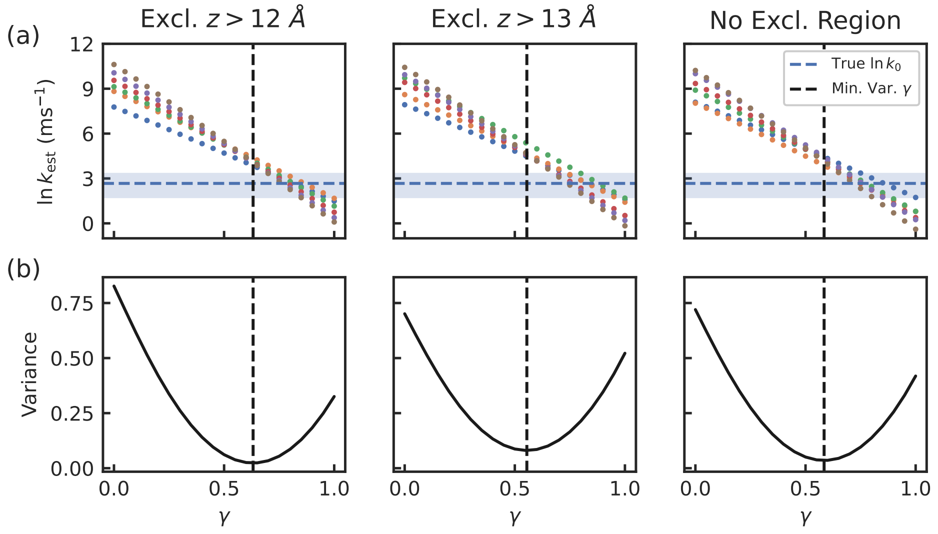

Figure S8: The results of EATRf on the new cavity-ligand model when biasing the CV $\xi = 0.87z + (\sqrt{1 - 0.87^2})\theta$ with various excluded regions in OPES. (a) The values of $\ln k_0$ predicted by Eq. 8 for each set of OPES simulations with each BARRIER parameter for various values of γ. (b) The variance in the predicted value of $\ln k_0$ across the sets of simulations. The dashed lines represent the minimum variance value of γ, which is the value that causes all sets to give the same value of $\ln k_{\text{est}}$, and the corresponding $\ln k_0^*$.

Table S3: Effect of an excluded region on estimated cavity dissociation rate constants.

**OPES-flooding**		
Data set	$\ln k_0^{\text{est}}$ (ms^{-1})	
Excluded $z > 12$	1.53 ± 0.22	
Excluded $z > 13$	1.74 ± 0.24	
No excluded	1.75 ± 0.21	
**EATR-flooding**		
Data set	$\ln k_0^{\text{est}}$ (ms^{-1})	γ
Excluded $z > 12$	4.08 ± 0.41	0.63 ± 0.04
Excluded $z > 13$	4.70 ± 0.43	0.56 ± 0.05
No excluded	4.18 ± 0.42	0.59 ± 0.05
unbiased:	2.67 [1.62, 3.44]	
